# Understanding Older Adults' Memory Distortion in the Light of Stereotype Threat

**DOI:** 10.3389/fpsyg.2021.628696

**Published:** 2021-03-10

**Authors:** Marie Mazerolle, Amy M. Smith, McKinzey Torrance, Ayanna K. Thomas

**Affiliations:** ^1^Laboratoire de Recherches Intégratives en Neurosciences et Psychologie Cognitive & MSHE Ledoux, CNRS, Université Bourgogne Franche-Comté, Besançon, France; ^2^Department of Psychology, Quinnipiac University, Hamden, CT, United States; ^3^Department of Psychology, Tufts University, Medford, MA, United States

**Keywords:** aging, stereotype threat, memory distortion, regulatory focus, executive resources depletion

## Abstract

Numerous studies have documented the detrimental impact of age-based stereotype threat (ABST) on older adults' cognitive performance and especially on veridical memory. However, far fewer studies have investigated the impact of ABST on older adults' memory distortion. Here, we review the subset of research examining memory distortion and provide evidence for the role of stereotype threat as a powerful socio-emotional factor that impacts age-related susceptibility to memory distortion. In this review we define memory distortion as errors in memory that are associated with gist-based errors or source misattributions. Whereas, some of the reviewed experiments support the conclusion that ABST should be considered in the context of age-related differences in memory distortion, others reported little or no impact of stereotype threat. These discrepancies suggest that the role of ABST, and socio-emotional processes generally, in age-related changes in memory distortion are less clear. In this review, we argue that ABST does play an important role in age-related changes in memory distortion. We present evidence suggesting that discrepancies in the reviewed literature may be reconciled when evaluated in the context of the leading theories about stereotype threat: the Executive Resource Depletion hypothesis and the Regulatory Focus theory. We also discuss how differences in methodology and participant characteristics can account for a priori contradictory results in the literature. Finally, we propose some recommendations for researchers and practitioners when assessing memory in older adults.

## Introduction

Traditionally, age-related influences on cognition have been considered from the cognitive and cognitive-neuroscientific perspective. However, more recently, researchers have begun to also consider the influences of socio-emotional factors as they impact age-related cognitive change. For example, it is now clear that aging changes life goals, inducing a positivity bias that influences memory and decision making (Thomas and Gutchess, 2020). Another example of such socio-emotional factors is stereotype threat. Stereotype threat occurs when stigmatized people are placed in a situation in which they are aware that their performance could confirm negative stereotypes about their social group. The fear of confirming negative stereotypes may undermine people's ability to perform well (cf., Steele and Aronson, 1995). For example, stereotype threat effects have been found when Black American students are asked to report their ethnicity before completing an intelligence test (Steele and Aronson), or when older adults complete a memory test in the same room as a younger adult (Kang and Chasteen, 2009). In these situations, stereotype threat acts as an acute stressor that induces physiological stress, cognitive monitoring processes, affective responses, and efforts to cope with these aversive experiences (Schmader et al., 2008).

In Western culture, older adults are stereotyped as being forgetful, leaving older adults (i.e., 65+ years old, Crews and Zavotka, 2006) at risk of experiencing stereotype threat. This is evident when researchers examine older adults' veridical memory, or accurate recollection. After confronting age-based stereotype threat (ABST), older adults have been shown to underperform on a wide range of veridical memory tasks (for a recent meta-analysis, see Armstrong et al., 2017; Barber et al., 2020). These results suggest that caution is needed when evaluating older adults' memory, both in the laboratory and in clinical assessment settings. In addition, these results suggest that, beyond cognitive and neural mechanisms, socio-emotional factors may also influence age-related differences in cognition.

Beyond its influence on veridical memory, understanding the impact of stereotype threat on memory distortion in older adults seems particularly important to ascertain whether these distortions are only a consequence of irreversible cognitive decline or may also be influenced by socio-emotional factors. Memory distortion encompasses gist-based errors where individuals falsely remember conceptually-similar information and source misattributions where individuals correctly remember some pieces of information, but incorrectly associate the remembered information with a specific instance. For example, an older adult may remember having taken their daily dose of medication, but misattribute the source, having retrieved a memory from the previous day. Literature clearly demonstrates that older adults are more likely to make source misattribution errors and more likely to remember events that never happened as compared to younger adults (Devitt and Schacter, 2016). Age-related differences in memory distortion have been investigated in two well-known paradigms—the Deese, Roediger, McDermott (DRM) paradigm (Deese, 1959; Roediger and McDermott, 1995) and the misinformation paradigm (Loftus et al., 1978). This review will focus specifically on how ABST impacts older adult performance in these paradigms.

Interestingly, ABST has also been shown to influence older adults' susceptibility to memory distortion. A small body of research suggests that older adults experiencing ABST may be more likely to demonstrate false memories in the DRM paradigm (Thomas and Dubois, 2011; Smith et al., 2017) and are less accurate within the context of the eyewitness misinformation paradigm (Thomas et al., 2020). However, the negative impact of stereotypes on older adult memory performance does not appear reliable, with studies failing to observe an impact of ABST on older adults' memory distortion (Henkel, 2014) or observing lower susceptibility to false memory under stereotype threat (Wong and Gallo, 2016).

By focusing on memory distortion as opposed to memory veracity, the present review offers novel and practical insights into the neural and cognitive mechanisms involved in ABST. Research on memory and aging suggests that age-related increases in memory distortion are associated with changes in the medial temporal lobes and prefrontal cortex (for a review see Devitt and Schacter, 2016). By characterizing the effects of ABST on memory distortion, we can hypothesize about how it independently impacts memory distortion and how it may influence neural functioning in older adults. Research on the cognitive mechanisms that underlie memory distortion generally focuses on retrieval-based deficits (for a review see, Pierce et al., 2003). For example, a false memory of purchasing apples at a grocery store may be attributed to a false sense of familiarity during retrieval, after having encountered several fruits at the store. By documenting whether ABST influences older adults' reliance on retrieval cues that may increase source or familiarity misattributions, this review may offer a path toward developing supportive retrieval-based strategies to reorient reliance on more diagnostic retrieval cues.

For this review, we identified empirical articles, meta-analyses, and reviews that investigated the effects of ABST on memory distortion in older adults. To find articles, we used the search terms *older adult(s)* or *aging* in combination with *stereotype threat* and *memory, episodic memory*, or *false memory*. After identifying articles that fit these criteria, we also reviewed the references from each article to determine whether additional articles were appropriate. For the purposes of the present review, we chose from these articles those that featured one or more dependent variables that measured memory distortion. This process yielded five articles (six experiments) that used either the DRM paradigm (cf., Deese, 1959; Roediger and McDermott, 1995) or an eyewitness-memory paradigm (cf., Loftus et al., 1978), and investigated stereotype-threat effects by either comparing older adult and younger adult performance or solely older adult performance (see Table 1). The examination of ABST on older adult memory distortion has been investigated only within the context of gist-based errors using the DRM paradigm and the eyewitness misinformation paradigm. Therefore, our review examines the research on ABST in the context of these two paradigms and proposes to reconcile seemingly conflicting results.

**Table 1:** Summary of the studies that examined the relationship between stereotype threat and memory distortion or episodic memory errors in older adults.

**Studies**	**Participants**	**Threat induction**	**Memory test type**	**Results**	**Additional measures**
Henkel (2014)Eyewitness	*N* = 100: 43 YA, 57 OA YA = 19.6 (18–22) OA = 78.2 (64–91) Years of Ed: YA:14 OA: 15.4	Threat (blatant) = Older adults typically made errors on the test + instruction to be accurate on the upcoming memory test. Control (subtle induction?) = upcoming memory test. Negative feedback = feedback indicating errors on the first test + instruction to be more accurate on the upcoming memory test.	20 forced-choice recognition about previously watched video (taken twice, before and after Induction)	Response change (between Test 1 and Test 2): greater response change for the negative feedback group than for either the stereotype-threat or control condition in YA and OA.	Confidence: no impact of instruction
Smith et al. (2017) DRM	*N* = 166: OA only OA = 70.59 (56–90)	High threat (blatant): Article about memory declines with age + upcoming memory test. Control (subtle induction?): upcoming memory test. Warning: article about the deceptive nature of the DRM paradigm. No warning: nothing was said.	Y/N recognition	OA under High threat demonstrated higher false recognition than OA in the control condition, regardless of whether they were warned. Years of education and employment status moderated stereotype threat effects in the warning-absent groups.	
Thomas and Dubois (2011) DRM	*N* = 128: 64 YA, 64 OA YA = 18.8 (18–22) OA = 69.8 (60–74) YA:12.3; OA: 15.2	High threat (blatant): Article about memory declines with age + upcoming memory test. Low threat: Article about language-processing research + upcoming test about language processing and verbal ability.	Y/N recognition + confidence	The difference between High threat and Low threat in false recognition was significantly greater in OA than in YA. OA under Low threat were less confident in their false memories than OA under High threat.	Lexical decision task (stereotype activation): OA under High threat demonstrated higher stereotype activation (faster RT) than OA under Low threat
Thomas et al. (2020) Exp. 1 Eyewitness	*N* = 123: 61 YA, 62 OA YA = 19.41 OA = 73.51 Years of Ed: YA:12.3; OA: 15.2	High threat (blatant): Memory declines with age. Low threat: Some types of memory do not decline with age.	Cued recall about the video and narrative	OA under high threat produced fewer correct responses on consistent and misleading trials than OA under Low threat. OA under high threat leave more answers blank than OA under low threat. OA under high threat made less commission errors on neutral trials than OA under low threat.	Operation span. Numerically lower performance under High threat.
Thomas et al. (2020) Exp. 2 Eyewitness	*N*=132: 66 YA, 66 OA YA= 20.73 OA= 72.59 Years of Ed: YA:13.1; OA: 16.3	High threat (blatant): Article about memory declines with age. Low threat: Article about some types of memory that do not decline with age.	Cued recall about the video Source monitoring test	No effect of threat induction on accuracy OA under Low threat were less accurate at attributing the source to items than OA under High threat.	
Wong and Gallo (2016) DRM	*N* = 168: 84 YA, 84 OA YA = 21.19 (18–30) OA = 74.64 (65–87)	High threat (blatant): Article about memory declines with age. Low threat: Article about language-processing research.	Y/N recognition	OA under High threat recognized fewer non-presented critical lures than OA under Low threat.OA under High threat recognized fewer studied words than OA under Low threat.	

### Scope of the Current Contribution

This narrative review aims to provide readers with up-to-date knowledge about a small body of research conducted on the impact of ABST on memory distortion in the DRM (Section 2 Older Adults and DRM False Memories) and the eyewitness-memory paradigms (Section 3 Older Adults and Eyewitness Memory). Then we discuss the underlying mechanisms that have been proposed to account for memory distortion in the two paradigms that have been used, and highlight how stereotype-threat effects in older adults may interact with these proposed mechanisms (Section 4 Cognitive and Motivational Processes Underlying Stereotype Threat). We also review moderating factors that have been shown to influence stereotype susceptibility in older adults and discuss how consideration of these factors helps account for discrepancies in the results from experiments on the topic (Section 5 Methodological Factors). As a byproduct of synthesizing the research on the topic, this review will present a path forward in reconciling the inconsistencies in this literature and understanding the complex relationship between stereotype threat and memory distortion in older adults.

## Older Adults and DRM False Memories

**Figure 1:**
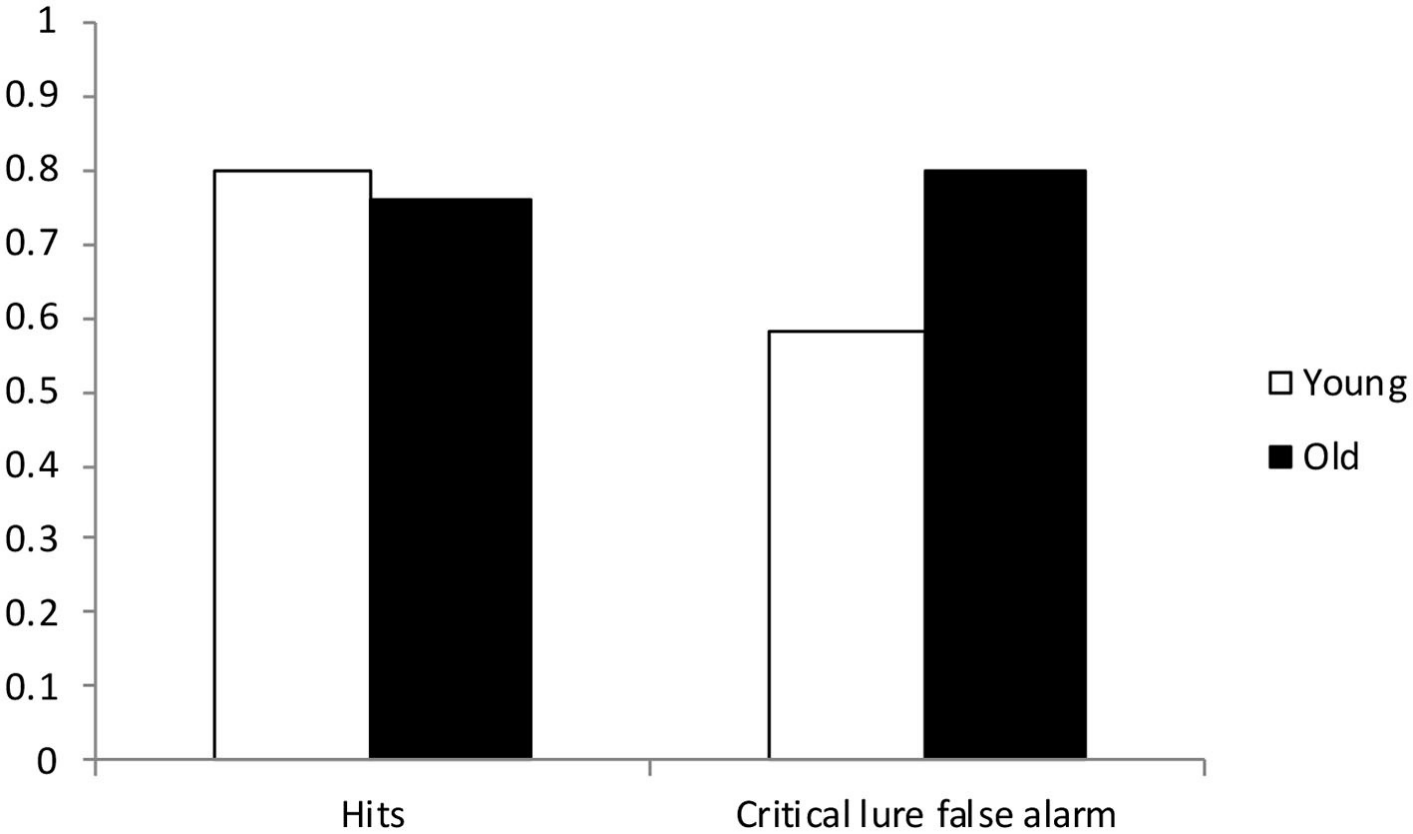
Mean proportion of Hits and Critical lure false alarms as a function of age group (*M*_age_ Young = 20.1; *M*_age_ Old = 70.7), adapted from Balota et al. (1999).

In the DRM paradigm, participants are presented with associated words (e.g., door, glass, pane, shade, ledge, sill, house, open, curtain, frame, view, breeze) and then perform a free recall or Y/N recognition test of memory. The typical finding is that people recall and/or recognize related but unstudied lure words (e.g., window) as having been previously presented (Deese, 1959; Roediger and McDermott, 1995; Mather et al., 1997; Robinson and Roediger, 1997; Balota et al., 1999). Older adults are generally more susceptible to these distortions in memory as compared to younger adults (see Figure 1, for an illustration of classic age differences observed in the DRM paradigm, adapted from Balota et al., 1999; see also Norman and Schacter, 1997; Tun et al., 1998; Kensinger and Schacter, 1999; La Voie and Faulkner, 2000; Dehon and Brédart, 2004). Research suggests that older adults may demonstrate increased false memory susceptibility in this paradigm because they are more likely to rely on relational processing as compared to younger adults (e.g., Thomas and Sommers, 2005). Relational processing, as compared with item-specific processing, is less likely to make individuating item information accessible at retrieval. Without this individuating information, people are less likely to effectively discriminate between targets and lures.

Item-specific processing involves the encoding of information that serves to individuate one item from another. This kind of processing focuses on the differences among items within a list and fosters accurate discrimination between studied items and test lures. In contrast, relational processing promotes the extraction of similarities across items. When engaging in relational processing, the learner often seeks to extract a shared cue that may be used to cluster individual items (Hunt and Einstein, 1981).

Research indicates that older adults may not be able to engage in item-specific processing as effectively as younger adults. For example, Kensinger and Schacter (1999) found that whereas younger adults reduced false memories across five study-test trials in the DRM paradigm, older adults continued to make similar levels of false recall and false recognition across the five study-test trials. These results suggest that younger adults may have used the accumulated contextual details acquired during repeated presentation of list items to decide whether information had or had not been presented. In another experiment, Thomas and Sommers (2005) presented DRM list items in the context of sentences to promote more individual-item elaboration. When sentences converged on meanings of words that were related to non-presented critical lures, older adults were more likely to incorrectly recognize the critical lure word as having been presented as compared to younger adults. In fact, encoding list items within the context of sentences reduced false memory susceptibility in younger adults, presumably because younger adults relied on the individuating contextual information afforded by sentences to discriminate between external presentation of a list item and internal activation of a lure. Importantly, Thomas and McDaniel (2013) later demonstrated that the reliance on relational as opposed to item-specific cues was more likely in older adults who scored low on a battery of tests that measured frontal functioning. These patterns have been interpreted as establishing that older adults, and specifically older adults with associated deficits in frontal functioning, are less likely to encode or retrieve item-specific information that can be used to effectively monitor the source of activated information, thereby distinguishing presented targets from non-presented lures (e.g., Smith et al., 2005; Thomas and Sommers, 2005).

Frontal function has been shown to moderate false memory susceptibility in older adults. Additionally, a small body of research suggests that ABST may also influence false memory susceptibility. For example, Thomas and Dubois (2011) found that older adults were more likely to falsely remember unstudied critical lures after stereotype threat was induced as opposed to reduced. In their experiment, older adults learned several DRM wordlists and then read either a passage about age-related changes in memory (i.e., High threat condition) or about language processing (i.e., Control condition). Afterward, they completed a recognition test, which consisted of studied items, unstudied items that were unrelated to the lists, and unstudied items that were the critical lures associated with each of the studied lists. As the first study to investigate ABST on memory distortion, Thomas and Dubois provided evidence that ABST may influence how older adults weigh cues at retrieval to decide between external presentation of a list item or internal activation. That is, under High threat, older adults may more likely rely on shared representations garnered from relational processing as opposed to individuating information. Reliance on shared representations, or taking a gist-based approach to recognition decisions, has been shown to be less cognitively demanding than relying on individuating features (e.g., Thomas and Sommers, 2005; Thomas and McDaniel, 2013).

With the publication of Thomas and Dubois (2011), several researchers attempted to better understand the relationship between ABST and memory distortion. However, pinning down that relationship and elucidating the underlying mechanism has proven challenging. Subsequent studies investigating ABST within the DRM paradigm have yielded results that either partially replicate or conflict with those reported by Thomas and Dubois.

Using a similar methodology to Thomas and Dubois (2011), Wong and Gallo (2016) presented participants with DRM list items for study and activated ABST prior to retrieval. As opposed to finding that ABST resulted in greater false memory susceptibility, Wong and Gallo found that it reduced false memory susceptibility. However, unlike in Thomas and Dubois, Wong and Gallo introduced a warning about the deceptive nature of DRM lists prior to retrieval. Warnings like the one used by Wong and Gallo are employed after encoding but prior to retrieval to isolate what Gallo and colleagues have termed “genuine” false memories. Importantly, pre-retrieval warnings do not eliminate false memory susceptibility (Gallo et al., 2001; Miller et al., 2011). Rather, these warnings are thought to reduce bias. Therefore, the stereotype threat effect on false memories in older adults under these conditions would represent an authentic change in memory as opposed to a change in response strategy. Yet, under these conditions, Wong and Gallo found that older adults who were exposed to the High threat induction manipulation were *less* likely to accept unpresented criterial lures as having been presented as compared to older adults who underwent the control manipulation.

Given that the use of the pre-retrieval warning was the only identifiable difference between the first two studies to investigate the relationship between stereotype threat activation and DRM false memories, Smith et al. (2017) examined whether the inclusion of the warning prior to test factored into the reduction of the stereotype threat effect observed by Thomas and Dubois (2011) by comparing four groups of older adults: ABST with a pre-retrieval warning, ABST with no warning, control with a pre-retrieval warning, and control with no warning. Importantly, they did not find a main effect of warning on DRM susceptibility or an interaction between warning and threat on DRM susceptibility. That is, warning did not moderate the impact of ABST on false memory susceptibility in older adults.

Though these three DRM experiments produced seemingly incompatible results, consideration of individual differences and guidance from the current theories about stereotype threat can help clarify the inconsistencies. Further, the DRM experiments hint at individual differences with false memory susceptibility (cf., Thomas and McDaniel, 2013). Considerations of individual-level differences in ABST will be discussed. But first, let us consider a second memory distortion paradigm in the context of stereotype-threat effects on older adults.

## Older Adults and Eyewitness Memory

**Figure 2:**
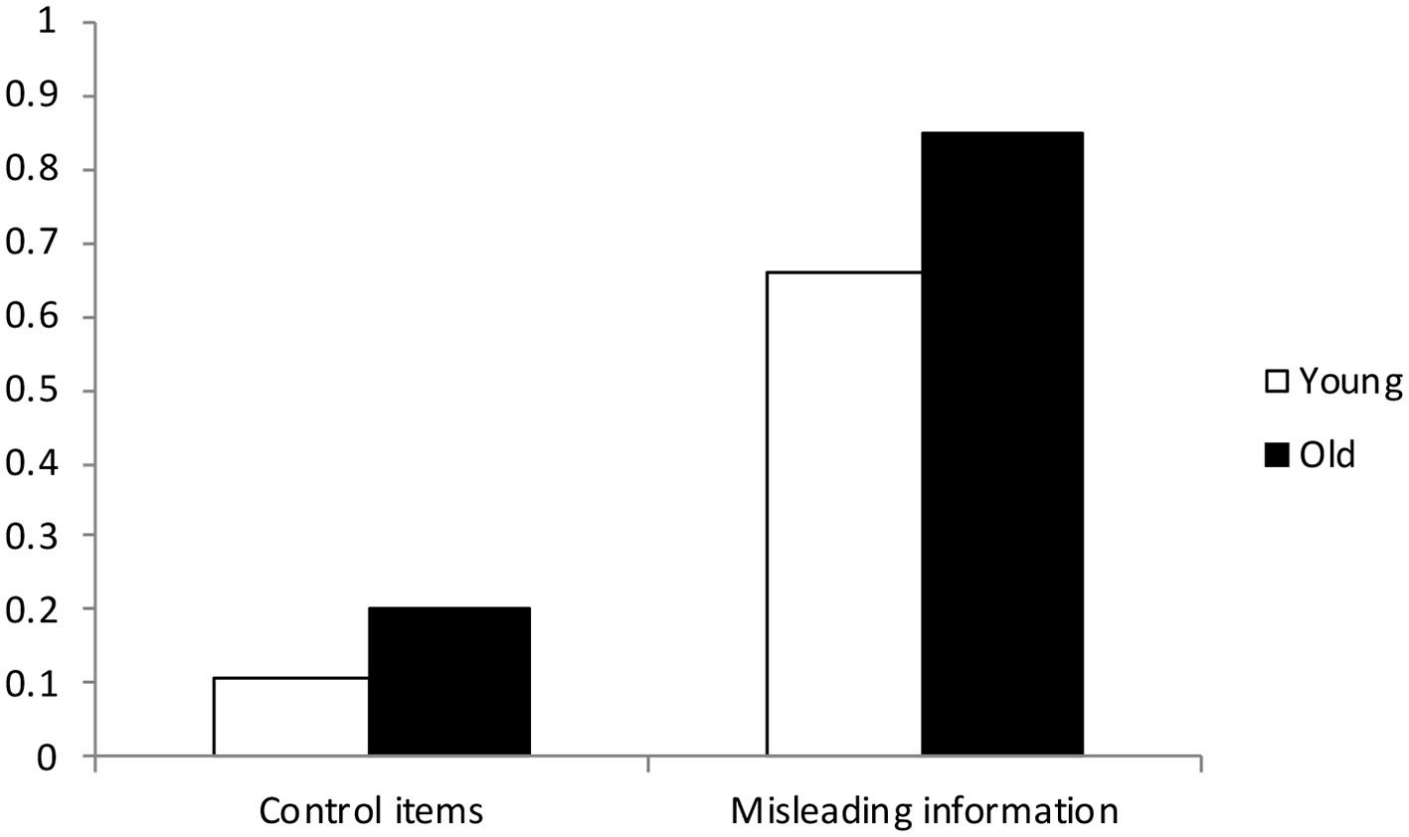
Mean proportion of *yes* responses for never-presented control items and misleading information as a function of age group (*M*_age_ Young = 19.6; *M*_age_ Old = 76), adapted from Mitchell et al. (2003).

In a standard misinformation experiment, participants witness an event and after some delay are exposed to misleading post-event information (Loftus et al., 1978). Misinformation can be presented in the context of a narrative of the original event or embedded in leading questions. Generally, researchers working in this area are concerned with the retroactive influence of the written synopsis or leading questioning on memory for the original event. Therefore, when participants are given the final memory test, they are asked to only respond with the original video information, and ignore information presented in the synopsis. Within this context, older adults have consistently demonstrated poorer performance for the control items (i.e., information only presented in the original event) and increased susceptibility to the post-event misleading information compared with younger adults (see Figure 2, for an illustration of classic age differences observed in an eyewitness memory paradigm, adapted from Mitchell et al., 2003; See also Cohen and Faulkner, 1989; Coxon and Valentine, 1997; Karpel et al., 2001; Bulevich and Thomas, 2012).

As with DRM false memory susceptibility, researchers have focused on understanding the factors that may impact successful discrimination between witnessed and suggested information. Unlike with the DRM methodology, the two sources of information are external (i.e., the witnessed event and the suggested post-event information). Research suggests that age-related increased misinformation susceptibility may result because older adults are less likely than younger adults to rely on effortful source monitoring that is necessary for accurate retrieval (see Thomas et al., 2014 for discussion). In support of this hypothesis, in a study with older-adult participants, Bulevich and Thomas (2012) found that a significant proportion of errors within the misinformation paradigm stemmed from problems at retrieval, and specifically with retrieval monitoring. Further, as with age-related DRM false-memory susceptibility, research has demonstrated that older adults who score lower on psychometric batteries designed to measure frontal lobe function are more likely to demonstrate memory distortion within the misinformation paradigm (Roediger and Geraci, 2007).

One common tool used to examine and promote effective retrieval monitoring is simply requiring participants to recollect the source of retrieved information using a source-monitoring test (cf. Johnson et al., 1993). In a source-monitoring test, participants are directly instructed to evaluate the source of a specific memory. Typically, this is done on an item-by-item basis. For every question, participants are required to attribute a source (original event or post-event narrative). The general finding from the misinformation literature is that source-monitoring tests reduce susceptibility to misleading information in both younger adults (Lindsay et al., 1991) and older adults (Multhaup et al., 1999), though the advantage gained by using a source-monitoring test is not consistently found with older adults (e.g., Mitchell et al., 2003).

In a recent study, Thomas et al. (2020) investigated the impact of stereotype threat on older adult memory in the eyewitness misinformation paradigm. They were interested in whether threat activation prior to retrieval would impact retrieval monitoring processes and conducted two experiments in which the nature of the final test demanded more or less retrieval monitoring. In Study 1, after the High or Low stereotype-threat induction procedure, the authors gave participants cued recall instructions that encouraged responding with information that came from either the video (i.e., the witnessed event) or the post-event synopsis. This task requirement was designed to reduce the need for retrieval monitoring. Additionally, participants took an Operation Span test to determine whether ABST impacted working-memory capacity. In Study 2, participants were required to respond with video information only. In addition, following the recall test, participants were presented with their answers and were asked to assess whether their answers really did come from the video or whether they may have been incorrect on the first test. In this case, participants were required to take two final tests, and both tests encouraged monitoring of retrieved information, directly and indirectly.

In Study 1, where retrieval monitoring was unnecessary for test accuracy, older adults in the High threat group were less accurate than older adults in the Low threat group. Importantly, the decrease in accuracy in the High threat group was directly related to errors of omission. That is, older adults in the High threat induction group were more likely to withhold responses than those in the control group. On the Operation Span test, ABST resulted in numerically lower performance; however, the interaction did not reach the level of standard statistical significance (*p* = 0.06). In Study 2, when encouraged to engage in retrieval monitoring, older adults in the High threat group did not differ from older adults in the Low threat group. Further, on a final source discrimination task, older adults in the High threat group were more accurate than those in the Low threat group.

The combined results of the two studies suggest that when negative stereotypes are activated prior to retrieval, older adults may approach a memory test that requires discrimination between two external sources of information in a more cautious manner than when threat is not activated. In Study 1, stereotype threat induction was associated with increased withholding. In Study 2, when asked to make source attributions, older adults under high threat demonstrated better accuracy than those under Low threat. These results suggest that ABST may foster a cautious approach to a memory test.

However, as with the DRM stereotype-threat literature, the small body of research focused on ABST and eyewitness memory has yielded inconsistent results. In another experiment using an eyewitness memory paradigm, Henkel (2014) had older and younger adults watch a short video clip and then gave participants a two-alternative forced-choice memory test that consisted of misleading and non-leading questions. For misleading questions, both options were incorrect. For non-leading questions, one option was correct and one was incorrect. A stereotype-threat induction procedure followed the first test. In the High threat condition, participants were told that people of their age group tended to get many answers wrong on the memory test, and that they would be asked to retake the test. In the Control condition, participants were simply told they would retake the memory test. A third group of participants were told that they had made several errors and should reconsider their answers when they retake the memory test. Afterward, all participants were given a second test, which was identical to the first. As would be expected, the negative feedback condition resulted in response change between the first and second memory test. More surprisingly, ABST did not lead to a statistically reliable change between the two tests, suggesting that stereotype threat did not cause older adults to doubt their memory.

Taken together, the few studies that investigated the impact of ABST on older adults' memory distortion (i.e., DRM and eyewitness memory paradigms) provide initial evidence of the important role of socio-emotional factors in age-related susceptibility to false memories. That is, facing a situation where negative age-related stereotypes are salient may have resulted in a stress response that had downstream consequences on memory. We argue that the socio-emotional impact on false memory susceptibility is relevant in understanding the mechanism that may underlie age-related changes in memory distortion. Specifically, socio-emotional processes may independently impact and/or directly interact with neurological changes in medial temporal lobes and/or the prefrontal cortex to increase or decrease memory distortion. That said, the six experiments discussed thus far present a puzzling picture regarding the impact of ABST on memory distortion. Though perplexing when taken at face value, we argue than when cognitive and motivational processes underlying ABST are considered along with methodological and participant-level differences, some clarity is gained and a path forward is uncovered.

## Cognitive and Motivational Processes Underlying Stereotype Threat

False memories in the DRM paradigm and decreased accuracy in the misinformation paradigm as a result of ABST have been considered within the context of the Executive Resource Depletion (ERD) account (Schmader et al., 2008). According to this account, stigmatized or threatened individuals are likely to engage in self-monitoring processes of performance during a stereotyped task. In conjunction, threatened individuals likely employ emotion-regulation strategies in order to manage negative thoughts and feelings associated with threat. Additionally, stereotype threat may trigger a physiological response to threat that impairs prefrontal processing (for a review, see Schmader, 2010). As a result of these complementary and simultaneous cognitive, physiological, and affective processes, threatened individuals may be left with fewer resources available to perform the targeted cognitive tasks, resulted in a cost in performance that is most likely demonstrated when tasks are complex. Consistent with this hypothesis, studies have demonstrated that ABST negatively impacts memory in older adults (e.g., Hess et al., 2003; Kang and Chasteen, 2009). Providing further support for the ERD account, ABST has been shown to reduce the efficiency of older adults' controlled processes (Mazerolle et al., 2012) and reduce performance on tasks that highly rely on working memory (Desrichard and Köpetz, 2005; Mazerolle et al., 2012; Swift et al., 2013; but see Hess M. T. et al., 2009 for contradictory results).

Returning to the literature on ABST effects on memory distortion, the studies presented earlier (See also Table 1) provided support for the ERD account. That is, Thomas and Dubois (2011) observed that stereotype threat increased DRM false memory susceptibility in older adults and argued that ABST further increased reliance on similarity among items as opposed to differences between items when making recognition decisions. Increasing reliance on individuating information and reducing reliance on relational information at retrieval is cognitively demanding and has been related to frontal functioning (cf., Thomas and McDaniel, 2013). As predicted by the ERD account, stereotype threat affects performance on the more cognitively-demanding tasks. This suggests that ABST depletes mental resources, which are already depleted by age, thereby resulting in a reliance on less cognitively-demanding processes.

Alternatively, studies reporting increased response withholding (Thomas et al., 2020) or reduced memory distortion (Wong and Gallo, 2016) when experiencing stereotype threat are consistent with a different account of stereotype threat effects: the Regulatory Focus hypothesis (RF, Barber and Mather, 2013b; Barber, 2017). This hypothesis predicts performance decrements when there is a conflict between the prevention focus instantiated by the activation of negative stereotypes (e.g., Seibt and Förster, 2004), and the promotion focus instantiated by traditional cognitive tests (e.g., Grimm et al., 2009), regardless of the cognitive demands of the task. Further, the RF hypothesis predicts that people should perform better on cognitive tasks when their internal regulatory state “fits” the task's reward structure. That is, performance is increased when people with a promotion focus (i.e., concerned with the presence or absence of gains) encounter a task in which they receive rewards (e.g., earning money for correct answers), or when people with a prevention focus (i.e., concerned with the presence or absence of losses, for example under stereotype threat) complete a task where they must avoid punishment (e.g., losing money for wrong answers).

Barber and Mather (2013b) provided a clear illustration of the RF in the context of ABST in older adults. In their experiment, older adults under stereotype threat demonstrated better recall performance when told that they would lose money for each word forgotten, but worse performance when told that they would receive monetary gains for each word correctly recalled. As more evidence for the RF hypothesis, older adults under stereotype threat have demonstrated risk aversion (Coudin and Alexopoulos, 2010) and increased accuracy and reduced speed on a test of working memory (Popham and Hess, 2015). When examining errors of omission and commission in a standard word-list paradigm, Barber and Mather (2013a) observed that ABST improved recall accuracy on a free-recall test (Study 1) and decreased false alarms, characterized by the adoption of a conservative response bias, on a recognition test (Study 2).

Although we have presented two different hypotheses to account for the apparently contradictory results obtained when examining the relationship between ABST and memory distortion, we suggest that ERD and RF are complementary and likely interact. Literature on RF clearly indicates that negative stereotypes fostered a risk-averse, vigilant processing style, that may improve or diminish performance as a function of the “fit” with tasks requirements, in particular in terms of intuitive vs. analytical thinking (Seibt and Förster, 2004). Interestingly, the ERD model also highlights the presence of monitoring processes to analyze self-performance. That is, under stereotype threat, individuals are more vigilant to threat and failure-related cues (Schmader et al., 2008). This idea is corroborated by studies demonstrating that stigmatized individuals demonstrate increased thoughts about monitoring their performance and its consequences (Beilock et al., 2007) and increased vigilance to task errors, as demonstrated by typical performance-monitoring error-related negativity pattern (Forbes et al., 2008).

When concurrently considering the ERD and RF accounts, it becomes likely that the two theories document the same phenomenon: increased vigilance toward errors and signs of failures under stereotype threat. In an effort to articulate the two hypotheses, it seems plausible that under stereotype threat, individuals would adopt a prevention focus that induces monitoring processes, and thus differentially taxes working memory resources depending on task structure (oriented to promotion/gain vs. prevention/losses). This possibility nicely fits the apparently contradictory results obtained on the DRM paradigm by Thomas and Dubois (2011) and Wong and Gallo (2016). In both experiments, participants were exposed to a blatant stereotype threat induction in which they read a scientific passage on age-related cognitive decline. This induction may have induced a prevention focus, orienting participants toward avoiding errors (Seibt and Förster, 2004). However, in the case of Thomas and Dubois (2011), the recognition test was presented without additional information. Because cognitive tasks, and maybe even more recognition tests, are by default oriented to promotion (Koriat and Goldsmith, 1994; Koriat et al., 2011), the “misfit” between older adults' prevention focus under ABST and the task (oriented to promotion) may have depleted older adults' executive resources and led to increased memory errors. On the other hand, Wong and Gallo clearly oriented their participants toward prevention, using a forewarning that emphasized avoiding memory errors. That is, participants may have experienced a “fit” between their prevention focus (induced by stereotype threat) and the task orientation toward avoiding false recognition that, in turn, improved their performance.

In the eyewitness memory paradigm, Thomas et al.'s (2020) results also nicely fit the idea that a prevention focus, as instantiated by task structure, negatively impacts working memory resources. The pattern observed by Thomas et al. in Study 1 under High threat (numerical decrease in working memory Operation Span and increased information withholding on the episodic memory test) is in line with the hypothesis that the ERD and RF accounts should be considered in concert when interpreting these results. Importantly, in Study 1, the task structure may have resulted in incongruency between task orientation (i.e., toward promotion) and the focus induced by ABST (i.e., toward prevention). In Study 2, the absence of stereotype threat effects on memory distortion combined with increased accuracy on a source monitoring task under ABST also aligns with this combined theoretical account. Explicit instructions to distinguish video from post-event synopsis information may have oriented participants toward prevention and led them to experience a “fit” with their prevention focus under ABST.

## Methodological Factors

When considering the discrepancies in the research on stereotype threat and memory distortion in older adults, there are additional methodological factors beyond the regulatory fit of the experiment that must be considered. As Table 1 demonstrates, there is a wide range of methodological differences across the small body of research examining ABST and memory distortion. In one experiment that highlighted the importance of these factors, an unusual style of threat induction was used, and ABST had no influence on memory distortion in older adults (Henkel, 2014). In another experiment, ABST only increased memory distortion in older adults when certain participant characteristics were considered (Smith et al., 2017). We suggest that, in addition to considering interactions between ERD and RF, stereotype threat effects on memory distortion should be considered within the context of threat-induction procedures and relevant participant characteristics.

### Type of Induction

Special attention must be paid to the methods by which researchers induce stereotype threat in older adults, as researchers employ a wide range of threat-induction procedures. Threat is sometimes induced using explicit methods such as those employed by Thomas and Dubois (2011) and sometimes through more indirect methods. Explicit inductions deliberately describe age differences and are characterized as blatant or fact-based inductions. Indirect or implicit inductions more subtly hint at age-based stereotypes, by, for example, mentioning that participants of different ages will be compared in a given experiment. Indirect inductions are characterized as subtle or age-based inductions.

Blatant manipulations are used in most research investigating the impact of ABST on older adults. These inductions present factual statements that inform older participants about their inferiority in memory/cognitive abilities and clearly define the task as a measure of memory/cognitive capacity. These inductions rely on salient cues, so that participants are aware of the stigma and the fact that their performance could validate the stereotype about their group. This kind of induction affects older adults' performance expectations but likely does not directly influence the threat caused by age-based stereotypes. For example, studies using blatant inductions informed participants about recent findings showing age-related decrements in memory performance (e.g., O'Brien and Hummert, 2006) or had participants read fake news articles that emphasized the negative effects of aging on memory (e.g., Hess et al., 2003, Wong and Gallo, 2016). Other studies induced stereotype threat by telling older participants that the study's objective was to evaluate memory—a cognitive domain known to decline with age (Bouazzaoui et al., 2016)—or informing older adults that younger adults usually outperform them on the memory test they are about to complete (e.g., Hess T. et al., 2009; Bensadon, 2010; Fernández-Ballesteros et al., 2015).

On the other hand, subtle inductions rely on less conscious processes, and are more indirect. Usually these inductions mention age differences in memory/cognitive capacity, but the direction of these differences is not explicitly mentioned. Even more subtle inductions simply mention age group membership and/or emphasize that the experiment involves evaluation of memory or cognitive abilities. Consistent with stereotype threat theory (Steele, 1997), when using subtle cues, older adults may feel “the threat in the air,” being reminded about age-related stereotypes while keeping ambiguity surrounding the stereotypes. For example, some studies informed older adults that they were about to perform a test that evaluates memory ability and had them complete the task in the same room as a younger adult (Kang and Chasteen, 2009) or simply mentioned to older adults that younger adults were also taking part to the study (e.g., Mazerolle et al., 2012; Wong, 2014).

The distinction between subtle/stereotype-based manipulations vs. blatant/fact-based manipulations has been recently addressed in two meta-analyses (Lamont et al., 2015; Armstrong et al., 2017). Lamont et al. (2015) highlighted that subtle manipulations (*d* = 0.49) had a greater impact on older adult memory and motor-task performance as compared to blatant manipulations (*d* = 0.16). The authors suggested that subtle manipulations may have a greater impact because they cause uncertainty about the presence of a threat. This ambiguity may increase distracting thoughts and tax cognitive resources required for successful performance on difficult tasks (Schmader et al., 2008).

Beyond the direct vs. indirect distinction in induction procedures, many stereotype threat studies vary in how they define their comparison baseline group. The way baseline groups are defined can potentially distort the conclusions based on non-observed stereotype threat effect (e.g., Shewach et al., 2019). When considering the baseline group in any older-adult stereotype-threat experiment, it is critical to consider whether subtle threat induction is unintentionally present. Subtle forms of threat induction may arise from apparently innocent statements, e.g., mentioning in an informed-consent form that the experiment is investigating the influence of age on cognition, and can have profound impacts on older adult memory performance (see Lamont et al., 2015; Armstrong et al., 2017), notably in a baseline group of older adults.

Returning to the literature on memory distortion and stereotype threat, and as Table 1 demonstrates, in the six experiments included in this review, threat induction was not held constant. Although all experiments employed a blatant threat induction for the High threat groups, the baseline comparison groups differed. Comparing performance between participants exposed to blatant vs. control induction will likely result in different conclusions than when performance for blatant and subtle threat induction is compared. To support this idea, Thomas and Dubois (2011), Wong and Gallo (2016), and Thomas et al. (2020) used control groups (i.e., exposing older adults to positive passages about aging and language processing) and found greater ABST effects in both false memory susceptibility and familiarity-based responding than experiments that compared High threat induction to subtle-threat induction (i.e., informing participants that a memory test was upcoming, Henkel, 2014; Smith et al., 2017). In Henkel's (2014) experiment, participants completed multiple memory tests and, after completing the first one, were assigned to one of the three experimental conditions. In the stereotype-threat condition, participants were told that people of their age group tended to get a lot of the answers wrong -a blatant induction- and would have to go through the questions again with the objective of being more accurate. In the control condition, participants were told that they would have to go through the questions again as a delayed recall task. One could speculate that the simple fact of completing a first memory test and being told that a second one was coming was sufficient to create a subtle stereotype threat induction (cf., Geraci and Miller, 2013; Rossi-Arnaud et al., 2018).

That said, threat induction methods do not account for additional differences in results across these experiments. We next consider how the demands of the memory test may interact with threat induction to influence ABST.

### Task Demands and Task Valuation

As mentioned in the previous section, the impact of age-related stereotypes on memory performance was reliable, with an average effect size of *d* = 0.26 (Lamont et al., 2015). Armstrong et al. (2017) further clarified the impact of blatant *vs*. subtle inductions, focusing on episodic and working memory tasks. In line with the Lamont et al. (2015) results, working memory was found to be more strongly impacted by subtle inductions (*d* = 0.96) than blatant inductions (*d* = 0.06). However, a stronger effect of blatant (*d* = 0.26) over subtle (*d* = 0.23) manipulations was found on episodic memory. This surprising finding should be interpreted with caution because of the small number of studies on which it relies (three studies), but also because each of the three studies rely on different episodic memory tasks (i.e., cued recall, recognition, and free recall). Using a subtle stereotype threat induction, Kang and Chasteen (2009) observed a significant effect of ABST on free recall (*d* = 0.68), but not on cued-recall or recognition. This finding is consistent with the general pattern that threatened or stigmatized individuals are at a higher risk of experiencing performance decrements on difficult tests because these tasks create greater physiological stress responses (Ben-Zeev et al., 2005) and also require more controlled processing (Schmader et al., 2008). In line with this idea, Nguyen and Ryan's (2008) meta-analysis clearly demonstrated that both racial/ethnic-based and gender-based stereotypes interacted with test difficulty, with more difficult tests producing larger effect sizes (*d* = 0.43 and *d* = 0.36, respectively). In the six studies examining the impact of ABST on memory distortion, we should note some variation in task demands associated with Y/N recognition tests, forced-choice recognition tests, and cued-recall tests (see Table 1). That is, it seems plausible that the modest impact of ABST sometimes observed in these studies may be, in part, due to the lower cognitive demand of those tests, as compared to free recall tests (cf., Bulevich and Thomas, 2012).

As with episodic memory, performance on working memory tasks in the context of ABST has produced inconsistent findings. These inconsistencies may be better interpreted when considering task demands and how the tasks may be interpreted by older participants. Some studies demonstrated reduced performance (e.g., Abrams et al., 2006; Mazerolle et al., 2012; Barber and Mather, 2013b; Swift et al., 2013 gain-based condition), whereas other studies found only limited evidence for reduced performance (Thomas et al., 2020) or failed to observe working-memory impairment under stereotype threat (Hess M. T. et al., 2009; Popham and Hess, 2015).

Interestingly, Hess and colleagues (Hess M. T. et al., 2009; Popham and Hess, 2015; but also Thomas et al., 2020) used computation or Operation Span tasks to assess working memory capacity and did not explicitly label the tasks as memory tests (e.g., the test was characterized as a “test of quantitative skills”). Recent literature suggests that stereotype threat effects are more likely to occur on tasks that are directly targeted by age-related stereotypes (Haslam et al., 2012; Barber et al., 2015). That is, Computation or Operation Span tasks that may look more like a math test than a memory test may not trigger age-related stereotypes in older adults.

Some evidence in line with this idea can even be found in Popham and Hess (2015). They examined the impact of stereotype threat both in older adults (threatened about memory) and younger adults (threatened about college majors: Engineering vs. others). As mentioned earlier, stereotype threat did not affect older adults' Operation Span performance. However, stereotype threat effects did clearly appear in younger adults. We suggest that the interaction between task interpretation and group valuation may be an important consideration. ABST effects only appear when the task seems directly in line with a stereotyped ability: a math test for students majoring in Engineering, but not for older adults, who are perhaps more threatened about their memory. Only one study using a memory-distortion paradigm tested the impact of stereotype threat on working memory (Thomas et al., 2020). Because Thomas et al. used an Operation Span test to assess working memory, one may speculate that older adults' interpretation of the task was closer to a math test rather than a memory test, and did not seem sufficiently related to ageist stereotypes to elicit ABST effects.

Interactions between type of memory test and type of induction may also account for differences observed across the experiments under consideration. However, without further research that directly examines this interaction, we cannot speculate as to how these methodological factors impact the relationship between ABST and memory distortion.

### Participant Characteristics

#### Age

Numerous experiments have found that the impact of ABST on older adult memory varies as a function of age. Researchers have reported greater threat-related memory impairment for older adults that just entered “old age” (adults in their 50s and 60s) as compared to their older counterparts (Hess and Hinson, 2006; Hess M. T. et al., 2009; Eich et al., 2014). This discrepancy may be, in part, due to differences in self-perceptions of aging. Older adults in their 60s may be particularly fearful of entering into old age and fearful of confirming the stereotype about memory loss. In contrast, older adults in their 70s may be aware that they clearly entered the “older adult” age group, thus endorsing the negative stereotype and no longer feeling threatened by it (Hess and Hinson, 2006). This hypothesis is supported by research showing that negative age-related stereotypes impact older adults' self-image (Levy, 2009) and are associated with negative cognitive and physical outcomes (Levy et al., 2002a,b; Levy, 2003). On the other hand, positive self-perceptions of aging are associated with longevity and other positive health outcomes in older adults (Levy et al., 2002a,b), and it seems likely that older adults who internalize positive age stereotypes will be less vulnerable to ABST. In line with this idea, Fernández-Ballesteros et al. (2015) showed that participants with negative self-perceptions of aging were the most vulnerable to stereotype effects on a free recall test.

In the context of the research reviewed here on ABST and memory distortion, the older adult samples from the six experiments had mean ages that ranged from 70 years old (Thomas and Dubois, 2011) to 78 years old (Henkel, 2014; see Table 1 for mean age and age ranges of each study). Importantly, cognitive-aging researchers have classified older adults between the ages of 65 and 74 years as youngest-old, those between ages 75 and 84 years as middle-old, and those aged over 85 years as oldest-old (Alterovitz and Mendelsohn, 2013). Further, chronological age has been associated with increases in memory distortion susceptibility. In line with previous findings in the ABST literature, the study that had the older sample of older adults with an average age range close to 80 and considered middle-old (Henkel, 2014) is one that did not find any impact of ABST. However, without directly testing the impact of chronological age on the magnitude of ABST effects, it is difficult to speculate more about this point. In their experiment on ABST using the DRM paradigm, Smith et al. (2017) investigated the moderating role of age and observed limited support for the relationship between age and stereotype threat. Additionally, the impact of participants' age was investigated in the Armstrong et al. (2017) and Lamont et al. (2015) meta-analyses and age was not found to influence the effect of stereotype threat on older adults' performance. However, many studies included in the meta-analyses had small samples sizes (approximately 24 subjects per condition) that may not have allowed for a reliable investigation of age differences.

Interestingly, Smith et al. (2017) found that retirement status, a factor clearly related to age (i.e., with increasing age, older adults are more often retired than employed), did significantly moderate the effects of stereotype threat on false memory susceptibility. More precisely, threatened older adults who were still employed demonstrated lower DRM errors of commission as compared to retired older adults. These findings are consistent with the idea that age, and perhaps other variables that correlate with age (i.e., retirement status), can influence whether older adults internalize a negative stereotype about aging and interact with stereotype threat effects. Retired older adults may endorse more negative views about aging (Kruse and Schmitt, 2006) and be more vulnerable to ABST, in line with Fernández-Ballesteros et al.'s (2015) results.

#### Education

Beyond age, retirement, or self-perception of aging, older adults' level of education has also been investigated as a moderator of the impact of ABST on older adults' memory performance. For example, Hess T. et al.'s (2009) study considered the moderating roles of age, education, and concern about being stigmatized in the effects of stereotype threat on memory performance. They observed that education moderated the impact of ABST, with lower predicted rates of recall for those with higher levels of education in the High threat group. In other words, expectations were related to performance, but these expectations were primarily affected by the threat manipulation in those with high levels of education. In contrast, Andreoletti and Lachman (2004) observed that ABST only affected performance for older adults with <4-years college degree. However, it should be noted that although some individual studies have demonstrated a relationship between education and stereotype threat, Armstrong et al.'s (2017) meta-analysis reported no impact of education on the relationship between ABST and working memory or episodic memory.

In the small set of research investigating the impact of ABST on memory distortion, the four studies that reported participants' years of education indicated a high average number of years of education (>15 years, see Table 1). The only study of these that explicitly tested the impact of education (Smith et al., 2017) found that highly educated older adults were particularly at risk of experiencing memory deficiencies that result from stereotype threat. That is, education, and its potential interaction with other methodological factors, may be an important factor to consider when evaluating older adults' susceptibility to false memory under ABST.

Although the research is limited, we argue that participant characteristics should be considered when interpreting results regarding the impact of ABST on older adults' susceptibility to memory distortion. Importantly, it seems likely that these variables interact with each other, but more research is needed to fully understand their impact on older adults' performance, especially in the context of memory distortion. Future researchers may consider the use of linear mixed models to obtain a more precise estimate of the effect of ABST on memory distortion in older adults. Linear mixed models can accommodate numerous fixed and random factors in a single analysis (West et al., 2014). For example, participant characteristics such as retirement status, level of education, and age could be included as fixed subject-level variables in a model examining the main effect of ABST on memory distortion. On the item level, this example of a linear mixed model would provide the added benefit of accounting for random effects due to item-level variability.

## Summary and Conclusions

The present review summarizes a growing area of research on the relationship between ABST and memory distortion in older adults. We offer evidence that stereotype threat has a complicated influence on memory distortion in older adults that may only be understood through careful consideration of methodological factors and participant characteristics. The results reviewed here also contribute to a more nuanced understanding of the cognitive underpinnings of memory distortion generally. As an alternative to the retrieval-based deficits usually proposed to understand the age-related differences on memory distortion (for review see, Pierce et al., 2003), the studies reported here demonstrate that ABST-related increases in memory distortion (Thomas and Dubois, 2011; Smith et al., 2017) may also result from increased response withholding (Thomas et al., 2020). Alternatively, other studies suggest that ABST may also decrease false memory susceptibility when warnings are provided about the deceptive nature of the task (Wong and Gallo, 2016). The arguments presented here suggest that these discrepancies can be reconciled when evaluated in the context of the leading theories about stereotype threat and the methodological and sample differences between experiments.

Some of the glaring inconsistencies in research on ABST and memory distortion in older adults may be explained by the combined contributions of ERD and RF. The research we have reviewed suggests that, when under stereotype threat, older adults will adopt a prevention focus in which they are hypervigilant about making errors. When this focus is met with a similarly-focused memory test, such as when they are warned to exercise caution on the test (Wong and Gallo, 2016), older adults do not demonstrate threat-related memory distortion. The match in the regulatory focus of the participant and that of the memory test may reduce the burden on executive resources, resulting in these null findings. However, when older adults are under stereotype threat and are given a memory test that encourages liberal responding by implicitly encouraging responses to all prompts, such as a standard cued-recall or recognition test (Thomas and Dubois, 2011; Smith et al., 2017; Thomas et al., 2020, Study 1), the mismatch between their internal state (prevention-focused) and the task at hand (promotion-focused) may tax executive resources and result in the observed memory distortion.

The nature of the threat-induction techniques that have been employed can also help account for the discrepancies in research on ABST and memory distortion in older adults. For example, Thomas et al. (2020) reported cautious responding for older adults under stereotype threat but Henkel (2014) found no changes in responding for older adults under threat. Henkel's paradigm featured two memory tests, the first of which could have primed older adults to be cognizant of their performance on the second test. Simply making older adults vigilant about their memory performance could induce subtle stereotype threat in the baseline group, potentially masking any effect of the intentional stereotype-threat induction.

As demonstrated by Smith et al. (2017), subtle sample differences may similarly contribute to differences in memory distortion under stereotype threat. Previous research examining the effects of ABST on veridical memory documented the moderating roles of age and level of education (Andreoletti and Lachman, 2004; Hess M. T. et al., 2009). Adding to their findings, Smith et al. (2017) found that older adults who were retired and/or highly educated were shown to be most vulnerable to DRM errors of commission. These results highlight the importance of considering participant characteristics related to self-perception when conducting stereotype-threat research.

Some guidance naturally follows from our review of the small body of literature on ABST and memory distortion in older adults. Future researchers should consider whether their memory task is focused on gains or losses. Researchers should test the hypothesis that task focused on gains will result in the finding that ABST negatively impacts performance, and a neutral or possibly positive effect may be found when the tasks focus on losses. Of further consideration is whether the procedures used during threat induction and the accompanying baseline task have internal validity. In other words, whether there are any aspects of the experiment that may subtly induce stereotype threat in the baseline group of older adults. Finally, researchers should consider the influence of participant characteristics (e.g., age, retirement status, level of education) and consider statistical approaches, perhaps involving linear mixed modeling, to account for this variability.

### Future Directions

The studies examining ABST on memory distortion in older adults provide evidence that socio-emotional factors may, at least in part, influence the cognitive processes that underlie memory performance. We suggest that ABST may result in behavioral changes as measured by response output (e.g., changes in memory distortion) independent of variability in neural-network recruitment and age-related neurological decline. That said, the relationship between ABST and neural compensation and coping is understudied and would provide much needed insight to understanding the contribution neural and socio-emotional factors that may underlie ABST broadly, and ABST in memory distortion specifically. More precisely, the concept of Cognitive Reserve (CR) postulates that individual differences in the cognitive processes or neural networks underlying task performance allows for variability and individual differences in both age-related cognitive decline and compensation and coping in the context of brain damage (Stern, 2002). The examination of CR in older adults has focused on whether older adults recruit different neural networks when completing cognitive tasks as compared to younger adults. While there is ample research to suggest that older adults may recruit different neural networks when completing specific memory tasks, there is little research focused on differential recruitment in the context of ABST.

In addition to providing practical advice for future research, our analysis of the literature on ABST and older adult memory distortion sheds light on real-world considerations for older adult memory. The studies discussed here suggest that ABST can leave older adults more vulnerable to memory distortion, but perhaps only under conditions in which there is a mismatch between their regulatory state and the type of memory task they must complete. For instance, in the case of an older adult who is insecure about her memory and must go before a court as an eyewitness, it could be helpful to frame the recall task to fit with the prevention focus she is likely experiencing. Instructing her to be careful to only report details that she is certain of (prevention focus) might result in better memory performance than instructing her to report as many details as she can remember (promotion focus). Additionally, in scenarios like this, it is important to consider whether the individual characteristics of the eyewitness may leave her more vulnerable to experiencing subtle ABST. A witness who is retired, under the age of 70, and/or highly educated may be more susceptible to stereotype threat, making it even more important to consider the framing of the questions that are asked of her on the witness stand. Of course, future research is needed to determine if the results observed in laboratory research extend to situations like this.

## Author Contributions

Project initiation and primary drafting of the manuscript were done by MM and AT. All authors contributed to manuscript writing, read and approved the submitted version.

## Conflict of Interest

The authors declare that the research was conducted in the absence of any commercial or financial relationships that could be construed as a potential conflict of interest.
